# A Heterogeneous Network Modeling Method Based on Public Goods Game Theory to Explore Cooperative Behavior in VANETs

**DOI:** 10.3390/s20061802

**Published:** 2020-03-24

**Authors:** Qiuhua Wang, Hao Liu, Xing Jin, Zhen Wang

**Affiliations:** School of Cyberspace, Hangzhou Dianzi University, Hangzhou 310018, China; wangqiuhua@hdu.edu.cn (Q.W.); 171270006@hdu.edu.cn (H.L.); wangzhen@hdu.edu.cn (Z.W.)

**Keywords:** public goods game, cooperative vehicular networking, rationality, altruism, Zealot

## Abstract

Cooperative vehicular networking has been widely studied in recent years. Existing evolution game theoretic approaches to study cooperative behavior in Vehicular Ad hoc Network (VANET) are mainly based on the assumption that VANET is constructed as a homogeneous network. This modeling method only extracts part attributes of vehicles and does not distinguish the differences between strategy and attribute. In this paper, we focus on the heterogeneous network model based on the public goods game theory for VANET. Then we propose a Dynamic Altruism Public Goods Game (DAPGG) model consisting of rational nodes, altruistic nodes, and zealots to more realistically characterize the real VANET. Rational nodes only care about their own benefits, altruistic nodes comprehensively consider the payoffs in the neighborhood, while zealots insist on behaving cooperatively. Finally, we explore the impacts of these attributes on the evolution of cooperation under different network conditions. The simulation results show that only adding altruistic nodes can effectively improve the proportion of cooperators, but it may cause conflicts between individual benefits and neighborhood benefits. Altruistic nodes together with zealots can better improve the proportion of cooperators, even if the network conditions are not suitable for the spread of cooperative behavior.

## 1. Introduction

With the development of vehicle networking and related technologies, Vehicular Ad hoc Network (VANET) has a great potential in our in-car experience to make travel safer and faster. To realize the promising properties of VANET, there have been worldwide endeavors from automobile manufacturers, universities and academic institutions to provide the communication capabilities for vehicles and transport infrastructures, enabling vehicle-to-infrastructure (V2I), vehicle-to-vehicle (V2V) and vehicle-to-pedestrian (V2P) communication, which are collectively referred to as vehicle-to-everything (V2X) communication. By leveraging vehicular sensors and V2X communication, vehicles are able to run safety-related applications including traffic reporting, environmental monitoring, distributed surveillance, and so on [1,2]. To achieve the effective operation of these applications, vehicles should act as intermediaries and participate in packets dissemination. However, forwarding packets for other vehicles would consume too much communication bandwidth, a rational vehicle who aims to maximize its own utility may not be reluctant to cooperate without incentives [3]. Subsequently, the network performance of VANET will be significantly decreased if many participating vehicles deviate from cooperation. To solve this problem, it is necessary to provide vehicles with some incentives or propose some auxiliary means to promote node forwarding.

Due to the constantly changing topology in VANET, tracking past behaviors of vehicles is not sustainable. The available information is limited to the interaction with the neighbors in the previous period, so the strategy made by vehicles must be spontaneous and shortsighted, and methods like reputation mechanism or virtual credit based on that information are not suitable tools for helping to induce desirable behavior. The model based on public goods games can effectively increase the proportion of cooperative vehicles in VANET, but with the increase of the network density, the connectivity continues to increase, and the cooperator proportion may decrease significantly [4]. Therefore, it is feasible to find a benefit compatible method.

Over recent decades, game theory has served as a general theoretical framework to model and analyze group interactions. In early times, it mainly focused on the optimization of algorithm [5], and improved vehicle cooperation through incentives mechanism or optimization of the utility function [6,7]. The method based on algorithm optimization depends on different parameters. To achieve the best condition, continuous learning is required, but the highly dynamic topology goes against this process. When vehicles have only limited neighborhood interactions, the local information often cannot make the network achieve the overall optimum. Evolutionary game theory, especially the public goods game theory, is characterized by its high adaptability to address the network topology that changes with time and has been widely studied to analyze the behavior of peer-to-peer networks in the presence of free-riding. [8]. Making full use of characteristics of PPG to promote cooperation, Banerjee et al. [9] presented a PGG-based framework for information dissemination in VANET and found the general parameters of packet forwarding including the mobility on cooperation diffusion. Shivshankar et al. [3] turned to message forwarding and proposed a distributed model, then applied it to explore the effect of network properties of VANETs on node’s cooperation. Ding et al. [4] considered the situation of vehicles leaving and joining freely, simplified the calculation of nodes’ payoff and improved the cooperation under high-density network conditions.

**Figure 1:**
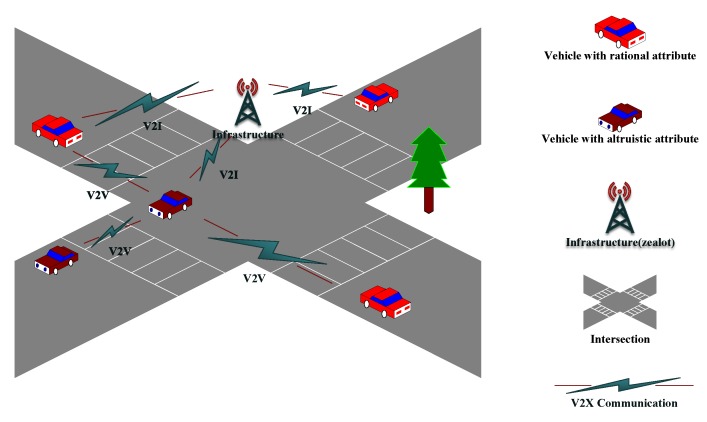
System architecture.

Previous works [3,4,9] mainly focused on applying PGG and its variants to the VANET and encouraged participants to forward packets. These works implicitly assumed that the survival of a cooperative strategy depends on the benefits achieved from the public pool in comparison with other strategies. Inspired by the combination of heterogeneous [10] networks and VANETs, we extend the modeling method proposed in the previous research and take the vehicle-to-everything (V2X) communication including vehicle-to-vehicle (V2V) and vehicle-to-infrastructure (V2I) into consideration. As illustrated in Figure 1, the network nodes are either static (i.e., transport infrastructures) or mobile (i.e., vehicles). Mobile nodes such as vehicles are divided into two types: rational and altruistic, while static nodes such as city-owned transport infrastructures remain as important intermediaries for packet forwarding under low network density. In such a heterogeneous architecture, both mobile and static nodes are equipped with V2I communication interfaces. The difference is that static nodes are also access to the control center, while dynamic nodes are available for V2V communication. The choice of which technology to employ for V2X communications is not restricted to a certain group of nodes but depends on the communication parties and packet types. In summary, the details of this article’s contribution are as follows:

- We take the attributes of nodes into consideration and then model VANET as a heterogeneous network containing nodes with rational and altruistic attributes. The previous works normally assumed that vehicles in the system are rational, i.e., each one aims to obtain more benefits through neighborhood interaction. However, many facts have proved that altruism is also an important internal attribute. When vehicles are communicating with each other, they will consider many external environment factors. Ignoring such an attribute of vehicles may lead to oversimplified or misleading conclusions [11].
- We introduce Other-regarding Preference (ORP) and fitness factor θ into PGG-based VANET model to more realistically characterize the altruism and consider the neighborhood in the assessment of nodes. Among the many external environmental factors, taking neighborhood benefits into consideration is the most common one. While making a strategy, they will consider not only their own benefits, but also the benefits of their neighbors.
- We define some city-owned infrastructures as zealots that always maintain a cooperative strategy in case of requests from neighborhoods. The previous works were mainly constructed on the communication of vehicle-to-vehicle (V2V). However, some transport infrastructures can also collect information from the surroundings and exchange this information in real time with other nearby entities. Unlike vehicles, infrastructure components such as RSUs usually have rich bandwidth, powerful computing capabilities, and abundant local storage. Therefore, these infrastructures can be regarded as a new type of node in heterogeneous vehicle networking.
- We construct an urban traffic road model and then evaluate the impacts of the different attributes on the cooperation under different network conditions. The introduction of altruistic nodes and zealots can significantly improve the proportion of cooperators, and the network can maintain a high level of cooperator proportion even under adverse conditions.

The rest of the paper is organized as follows. In Section 2, related works are discussed. In Section 3, a dynamic model with altruistic nodes and zealots is proposed in VANET. In Section 4, extensive simulations are conducted to illustrate the performance of the proposed model. Finally, the conclusion is drawn in Section 5.

## 2. Related Works

Sharing a similar motivation as ours, early studies on VANET mainly focused on incentive and punishment mechanisms. Li et al. [12] defined the measurement of contribution according to the unique characteristics of VANET, then proposed a secure incentive scheme to motivate vehicles and their drivers to cooperate in the process of packet forwarding. Chen et al. [6] presented a credit-based incentive system named MobiCent. This system used a Multiplicative Decreasing Reward algorithm to calculate payment, which can effectively foster cooperation among selfish vehicles with bounded overheads. Ng et al. [7] introduced the theory of imperfect private monitoring for Dynamic Bertrand Oligopoly in economics to promote cooperation by monitoring network nodes and punishing selfish nodes. However, there are still many shortcomings in these incentive-based schemes. Most of these schemes use the repository to track the past behavior of the nodes, or require credit exchange to limit the behavior of the nodes. VANET itself is a highly dynamic network, and the network topology varies greatly at different time instants. With the increase of network density, the storage and retrieval of vehicle information will consume huge computing resources, making it difficult to build a scalable system.

Game theoretic approaches have also been frequently used in packets dissemination models to discourage their selfish behaviors in various wireless networks [13,14]. For example, evolutionary game theory was applied to the access to the road-side unit and multimedia data dissemination [15]. However, in the process of group interaction, vehicle behavior tends to defect rather than cooperate when the game reaches the Nash equilibrium (NE). To address this issue, the coalitional game (CG) is commonly used to induce cooperative behaviors. Kapade et al. [16] proposed a trust point system based on the coalitional game for message forwarding and the load balancing problem. Chen et al. [17] proposed an end-to-end scheme based on coalitional game theory (CG) to stimulate message forwarding in VANETs. Zhang et al. [18] considered an advertisement distribution scenario and proposed an advertisement distribution scheme which applied coalition games to guide the sharing mechanism among private vehicles.

Public goods game is a standard model characterized by its prevalence of group interactions, which is a competitive tool for the study of cooperation in the wireless network and currently has been widely studied in social and computing networks [19]. All nodes in this model concentrate on reaping benefits from a shared public pool, and the benefit available to a particular group is assigned equally to all group members. Banerjee et al. [9] presented a PGG framework for content downloading in VANETs, and explored the impact of vehicle mobility on cooperation under this model. Shivshankar et al. [3] presented a PGG-based group interaction model for message dissemination and explored the impact of network attributes on cooperative behavior. Considering that nodes can choose to join or exit the game freely, Ding et al. [4] first presented a dynamic member public goods game model for real-world vehicle environments in which games are carried out within each group, then proposed a dynamic grouping public goods game model to model VANETs with high network density.

Most optimizations to existing models are based on the assumption that VANET is constructed as a homogeneous information network. As shown in Table 1, various models are developed to explore the group interaction among vehicles. These works implicitly assumed that nodes in the network all have the same attribute and maximize their own benefits through group interactions. However, this is not true, VANET is usually composed of many different types of nodes such as vehicles, transport infrastructures and so on. Nodes with different attributes play different roles, accordingly, using a single attribute to measure all nodes may cause information loss. Making full use of PPG’s high adaptability to network topology, we introduce altruistic nodes and zealots into the PGG-based VANET model and propose a Dynamic Altruism Public Goods Game model (DAPGG) to better improve the proportion of cooperators, and then explore the impacts of heterogeneous attributes on packet forwarding. As a heterogeneous network, its advantage lies in compatibility: by establishing an effective assessment of revenues, our work can coexist well with previous works.

## 3. Proposed Model

### 3.1. Packet Forwarding Game in VANET

References [3,9] presented a decentralized and structured V2V model based on the public goods game. "Decentralized" means that the system does not have a central agency to record the real-time conditions of each vehicle, "structured" means that vehicles in the system can only interact with their neighbors. At a given time slot, several packets are transmitted to the network through source nodes, which are initially set as cooperators. All vehicles in the model are willing to receive these packets; however, only cooperators take part in forwarding. At the beginning of each round of game, each cooperator is able to initiates a public goods game and only its direct neighbors can take part in this game. During the process of group interaction, a cooperator can be expected to make contributions to all its neighbors with a certain cost. Considering the total cost of forwarding a packet in a single slot as a fixed value c, all cooperative neighbors are contending to gain benefits from this transmission. Hence, to some extent, the cost of a cooperator can be regarded as the probability of packet forwarding in each time slot with respect to the number of cooperators in the neighborhood. Considering a fair random scheduling of nodes, the cost incurred by a cooperator can be formulated as:

(1) $$\eta_{i}=\frac{c}{n_{i}^{c}+1}$$

where $n_{i}^{c}$ denotes the number of cooperative neighbors. In the case of information dissemination, a packet received by a node makes sense if and only if it has not been received earlier. Thus, the total benefits obtained by a node in a single slot can be computed as the aggregation of investment from all neighboring cooperators, and can be given as:

(2) $$b_{i}=\frac{r}{n_{i}^{c}+1}\underset{j\in N_{j}\cup i}{\sum }\frac{c}{n_{j}^{c}+1}*t_{j}$$

where $N_{j}$ denotes the number of the direct neighborhood of node *j*, $t_{j}=1$ if *j* is a cooperator and has at least transmitted one packet which has not been received by *i*, and $t_{j}=0$ otherwise. A normalization factor $\frac{r}{n_{i}^{c}+1}$ is introduced to take the impact of neighborhood contention into consideration. For a PGG centered at node *i*, the payoff of a cooperator and a defector in a round of game are obtained as follows, respectively.

(3) $$\pi_{i}^{C}=b_{i}-\eta_{i}$$

(4) $$\pi_{i}^{D}=b_{i}$$

where $\pi_{i}^{C}$ and $\pi_{i}^{D}$ denote the payoff of a cooperator and a defector, respectively.

### 3.2. Dynamic Altruism Public Goods Game in VANET

All of the works above are based on the assumption that VANET is constructed as a homogeneous information network. However, most systems in real life are composed of various types of components interacting with each other. This modeling method extracts part of the attributes and does not distinguish the differences between strategies and attributes in the real system which usually results in incomplete information or information loss. Existing models implicitly assume that nodes are completely rational and maximize their payoffs by interacting with neighbors [3,4,9]. Many facts have proved that altruism is also an important internal attribute of nodes, and should be explicitly considered when modeling vehicles in the network [20,21]. Therefore, in this paper, we introduce Other-regarding Preference (ORP) into the PGG-based VANET model to more realistically characterize the altruism of vehicles and consider the influence of neighborhoods on the individual assessment.

Based on the above consideration, we propose a Dynamic Altruism Public Goods Game (DAPGG) model for VANETs to capture the interaction among different types of vehicles. In this model, nodes still can initiate games and participate in the game initiated by their direct neighbors at the same time. In the proposed DAPGG model, all nodes are divided into two categories: rational nodes and altruistic nodes, and update their strategies by comparing their revenues with a direct neighbor. Accordingly, different types of nodes have different revenue calculation methods. The rational node only cares about its own payoff and constantly strives for a higher benefit in the interaction with its neighbors, so the revenue only includes its own payoffs. Unlike the rational node, we use Other-regarding Preference (ORP) to calculate the revenue of altruistic nodes. More precisely, the altruistic node’s revenue not only includes its own benefit but also the average benefit of its neighbors. We can use $\pi_{i}^{\prime }$ to represent the mean payoff of neighboring nodes of node *i*:

(5) $$\pi_{i}^{\prime }=\frac{\sum_{x\in N_{i}}\pi_{x}}{|N_{i}|}$$

where $N_{i}$ denotes the immediate neighborhood of node *i*, and $\pi_{x}$ is node $i^{{}^{\prime }}$ s payoff which can be calculated by Equation (3) or Equation (4). To comprehensively consider the benefits of itself and the neighbors, the revenue of node *i* can be expressed as:

(6) $$U_{i}=\left(1-\theta \right)*\pi_{i}+\theta *\pi_{i}^{{}^{\prime }}$$

where θ∈[0,1] denotes the fitness factor, which is responsible for adjusting the degree to which the node attaches importance to the payoff of neighboring nodes in this round of the game. As θ decreases, less average payoff of its neighbors is considered, especially when θ=0, node *i* can be regarded as a rational node. The detailed game algorithm of a node is described by Algorithm 1.
**Algorithm 1:** DAPGG Algorithm
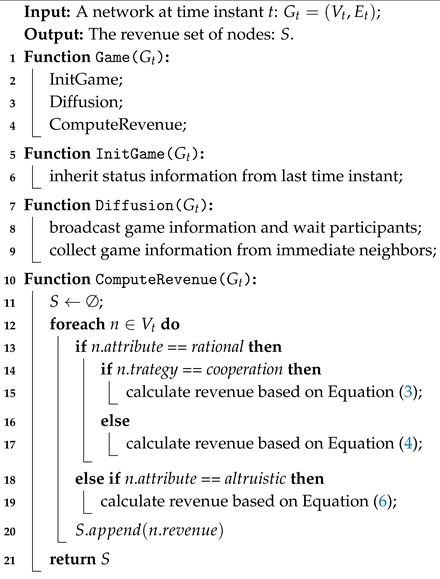


### 3.3. Stubborn Decision-Makers in DAPGG

Previous works [3,4,9] have primarily explored the cooperation of vehicle-to-vehicle. However, transport infrastructure is also an important component of vehicular communication systems. By collecting information from neighboring vehicles, these infrastructures can exchange information with surrounding vehicles in real time. Road-Side Unit (RSU) is a communication node installed within the infrastructure [15,22]. Taking RSUs into consideration while modeling, two benefits are provided: (1) RSUs can provide Internet access to vehicles allowing them to obtain with the emergency services immediately, thereby reducing notification times in case of an accident; (2) RSUs can rebroadcast packets delivered by vehicles in low vehicle density scenarios, allowing packets to arrive in more vehicles. To better model real-world scenarios, we introduce RSUs into our model.

As a new type of node in VANET, RSU is not limited to its volume and power. Therefore, RSUs can equipped with rich bandwidths, powerful computing capabilities, abundant local storages etc., and do not worry about the overwhelming load of services for others. Facing requests from neighbors, RSUs can keep cooperative strategy unchanged. In evolutionary games, this type of stubborn node that always maintains cooperative strategy is called Zealot [23,24,25]. In this paper, we integrate zealots into our DAPGG model, and then look into how the presence of zealots affects cooperative behaviors in VANET. The results are presented in Section 4.3.

### 3.4. Communication in DAPGG

In such a structured and heterogeneous network, how to coordinate the communication among different types of nodes still remains an open and challenging issue. Vehicle-to-everything (V2X), including vehicle-to-vehicle (V2V), vehicle-to-infrastructure (V2I), vehicle-to-pedestrian (V2P), and vehicle-to-network (V2N) communications, has become a research hotspot to address vehicular communication problems in the domain of VANET. By real-time sensing of the surrounding conditions of vehicles, V2X together with various sensor devices has a great potential of enabling a variety of safety-related applications. Today, V2X communication is mainly based on the following technologies: Dedicated Short Range Communications (DSRC) [26], Cellular-based V2X (C-V2X) [27] and DSRC-Cellular Hybrid Communications technologies [28]. In this paper, we mainly focus on the system-level communication architecture rather than the specific communication technology implementation. Since our model is composed mainly of rational nodes, altruistic nodes, and zealots, communication among different types of nodes also adopts different communication technologies. Specifically, V2V communication technologies are applied among vehicles with rational and altruistic attributes, and V2I communication technologies are adopted between vehicles and infrastructures.

When the node moves, the neighborhood of each node is determined by its communication range. However road-side buildings, road-side trees, large vehicles, road facilities, and so on, will affect the communication quality between vehicles and RSUs. Moreover, the wireless signal transmission will produce reflection, refraction, interference and other phenomena, these influences can cause the wireless channel quality to be unstable. In this regard, we use the quasi unit disc model (QUDM) [9] to determine the neighborhood of a given node and the probability $P_{C}$ to find a neighboring node is given relative to their distance dis,

(7) $$P_{C}\left(x\right)=\left\{\begin{array}{cc} 1 & \;\mathrm{if}\;dis<R_{in}\\ 1-{\left(\frac{R_{out}-dis}{R_{out}-R_{in}}\right)}^{\gamma } & \;\mathrm{if}\;R_{in}\leq dis\leq R_{out}\\ 0 & \;\mathrm{if}\;dis>R_{out} \end{array}\right.$$

where $R_{in}$ and $R_{out}$ denote internal and external communication radius, respectively. γ denotes the tuning factor between [0,1]. Determining the signal attenuation caused by reflection, refraction, interference, etc. requires expensive calculations in real time. QUDM provides a way to simplify noise in the surrounding environment, and thus helps to reduce the computational burden.

### 3.5. Evolution of Strategy

As with any VANET model in the real world, neighborhood information cannot be fully perceived. Therefore, with the change of neighborhood topology, game participants cannot make the best or most rational strategy in advance. Existing studies on evolutionary games, thus, offer a way to gradually improve the revenue through continuous strategy evolution. At a given time instant, each node re-examines whether to update its strategy via an imitation process that is based on the revenue of itself and its neighbors for the next round. As stubborn individuals will keep the cooperation strategy unchanged regardless of their own payoffs and the benefit of their neighbors, this strategy evolution process excludes stubborn individuals. In contrast to strategies, rationality and altruism are inherent attributes of the node and will not change with evolution. After each round of play, node *i* updates its strategy by comparing its revenue with a randomly chosen neighbor *j*, then node *i* adopts the strategy of neighbor *j* with a fermi-like probability obtained by Equation (8):

(8) $$P_{ij}=\frac{1}{1+\exp[\frac{R_{i}-R_{j}}{k}]}$$

where $R_{i}$ denotes the node *i*’s revenue, and *k* denotes the selection pressure (the sensitivity of nodes to the difference in the revenue). For k→+∞ (weak selection pressure), the probability approaches $\frac{1}{2}$ asymptotically, regardless of their revenue difference. On the other hand, for k→0 (strong selection pressure), nodes definitely update the strategy to gain more revenue. According to previous studies [3,29], *k* is usually fixed as 1. The detailed strategy update algorithm is described by Algorithm 2.
**Algorithm 2:** Strategy Update Algorithm
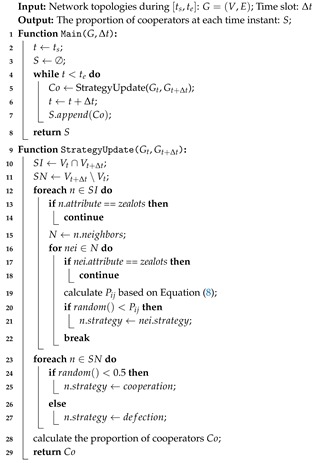


### 3.6. Mobility in DAPGG

In a highly dynamic network topology, mobility has a great impact on the node’s strategy evolution [9]. To get closer to the real scene, all nodes in our model are simulated in accordance with the Intelligent Driver Model (IDM) [30]. In traffic flow modeling, the IDM describes the dynamics of the position and velocities of single vehicles. For vehicle α, $x_{\alpha }$ and $v_{\alpha }$ denote its position and velocity at a given time instant *t*, respectively. The dynamics of the vehicle α are then obtained by the following ordinary differential equations:

(9) $$x_{\alpha }^{\prime }=v_{\alpha }$$

(10) $$s^{*}\left(v_{\alpha },\Delta v_{\alpha }\right)=s_{0}+v_{\alpha }T+\frac{v_{\alpha }\Delta v_{\alpha }}{2\sqrt{ab}}$$

(11) $$v_{\alpha }^{{}^{\prime }}=a\left(1-{\left(\frac{v_{\alpha }}{v_{0}}\right)}^{\delta }-{\left(\frac{s^{*}\left(v_{\alpha },\Delta v_{\alpha }\right)}{s_{\alpha }}\right)}^{2}\right)$$

where $x_{\alpha }^{{}^{\prime }}$ and $v_{\alpha }^{\prime }$ denote the new position and velocity, respectively, where $v_{0}$ is the velocity the vehicle would drive on a free road, *T* is the desired safety time headway to the vehicle in front, $s_{0}$ is the minimal distance to the vehicle in front, *a* is the acceleration, and *b* is the comfortable braking deceleration, together they determine the stability of the IDM model.

## 4. Simulation

### 4.1. Experiment Settings

**Figure 2:**
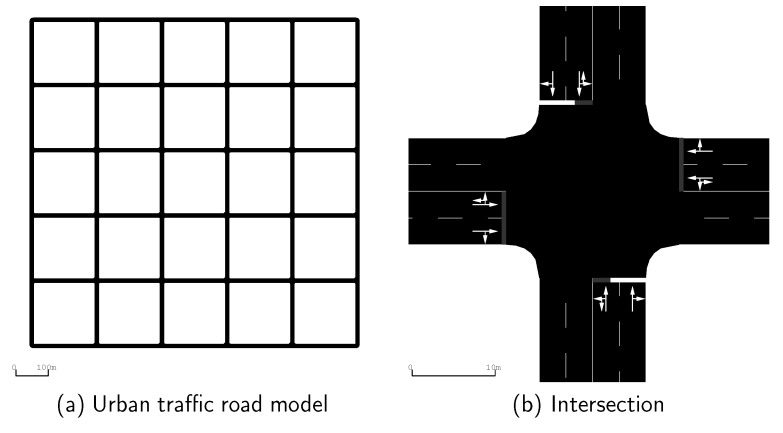
Simulation environment.

To explore the impact of our DAPGG model on cooperation in VANETs, we use SUMO [31] to construct a 1000 (m) × 1000 (m) urban traffic road model with N (50, 100, 200) vehicles. There are 6 × 6 bi-direction roads with two-way traffic and 36 intersections in the map. The distance between each parallel road is greater than 200 m, exceeding the vehicle communication range which is fixed as 100 m. Figure 2 shows a part of the above simulation environment.

**Figure 3:**
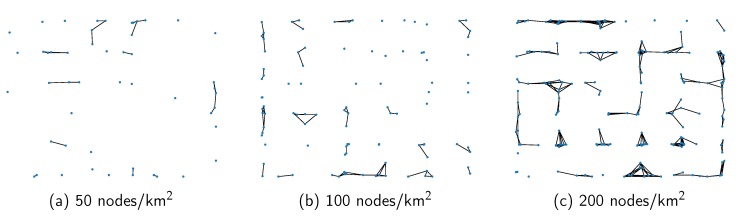
Network connectivity with different node densities.

Here, all nodes are simulated in accordance with the IDM. Figure 3 presents the network topologies under different node densities. At the beginning of the simulation, each node is initially set as a cooperator or defector with 50% probability. If the vehicle is a cooperator, it will forward the packets it receives from the neighbors, otherwise it will take the free ride of neighbors. In contrast to [3,9], we abandon the setting of source nodes. Each cooperator in our model can forward packets generated by itself or received from neighbors, which makes real-life VANETs more realistic. Subsequently, at the end of each time instant, the node updates its strategy based on the calculated revenue as described in Equations (3), (4), and (6). Table 2 shows all simulation parameters.

### 4.2. Altruistic Nodes in DAPGG

#### 4.2.1. The Impact of Simulation Numbers

**Figure 4:**
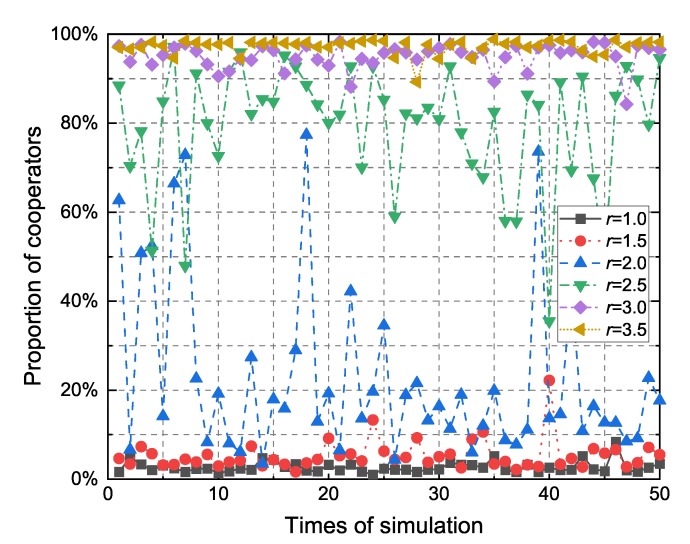
Proportion of cooperators for different synergy factors with respect to different simulation times (up to 50).

At the beginning of each simulation, most nodes cannot participate in forwarding because they may not generate packets or receive packets from neighbors which are stored by caches, so the proportion of cooperators in the early period is relatively unstable. In this paper, we record the average proportion of cooperators within 300–1200 s and take this value to represent the proportion of cooperators of the model during a simulation. Figure 4 shows the proportion of cooperators for different synergy factors with respect to different simulation times(up to 50). When the synergy factor *r* is less than 2.0, the proportion of cooperators stays in a lower range. When the synergy factor *r* is greater than 2.5, the proportion of cooperators remains in a larger range. However, the proportion of cooperators would fluctuate greatly when the synergy factor *r* is between 2.0 and 2.5. The result shows a sharp shift from a network state where almost all the nodes tend to defect to a state where almost all of them have a tendency to cooperate.

Generally, the model based on the public goods game shows that for a high-value synergy factor *r*, the proportion of cooperators seems to be the dominant behavior. In a structured network, the proportion of cooperators would be effectively increased due to the formation of the cooperator cluster. However, VANET is characterized by its highly dynamic network topology, which will quickly break the cooperator clusters. Therefore, it is more difficult for cooperators to survive in the highly dynamic network topology when the synergy factor is low (*r* <= 2.0). However, when the synergy factor exceeds a certain threshold (*r* >= 2.5), the cooperator can gain more payoffs than the defector as the benefit from the public pool is greater than the actual cost. In a word, as shown in Figure 4, there is a sharp shift from defection to cooperation with the increase of *r*. When the synergy factor *r* is close to the threshold (2 < *r* < 2.5), due to the uncertainty of the network topology, the proportion of cooperators in the network is also uncertain, causing a large fluctuation.

#### 4.2.2. The Impact of Synergy factor

**Figure 5:**
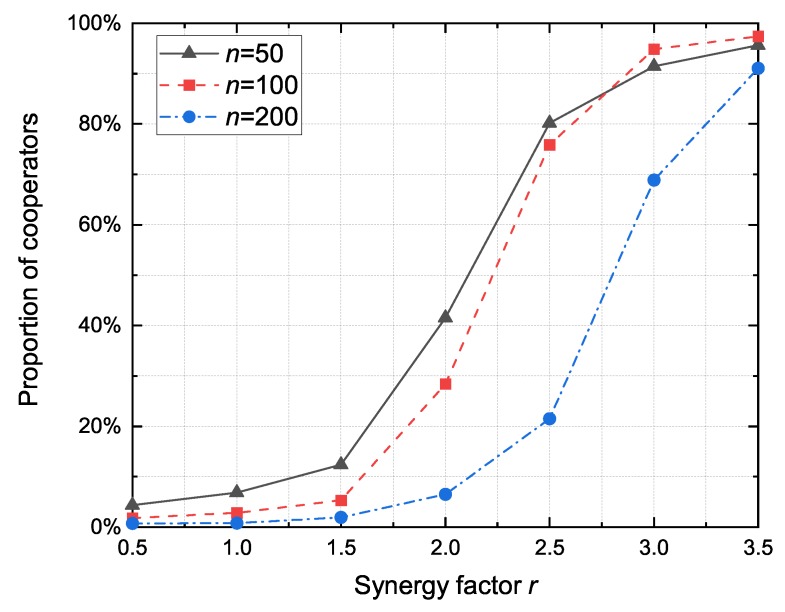
Comparison of the proportion of cooperators under different vehicle numbers for varying the synergy factor *r*.

We present the proportion of cooperators under different synergy factors with 50, 100 and 200 nodes in Figure 5. Under the same synergy factor *r*, it can be observed that the proportion of cooperators decreases as the number of nodes increases. The reason is that higher network density induces higher network connectivity, as shown in Figure 3. Because of the considerable number of advanced nodes, neighboring nodes share similar neighborhood structures. The cooperators contribute a certain cost to the public pool, while the defectors take the free ride of cooperators in the neighborhood. As a result, defectors with a higher payoff may cause neighbors to turn to defect, which will lead to a decline in the proportion of cooperators.

#### 4.2.3. The Impact of Altruistic Node Proportion

**Figure 6:**
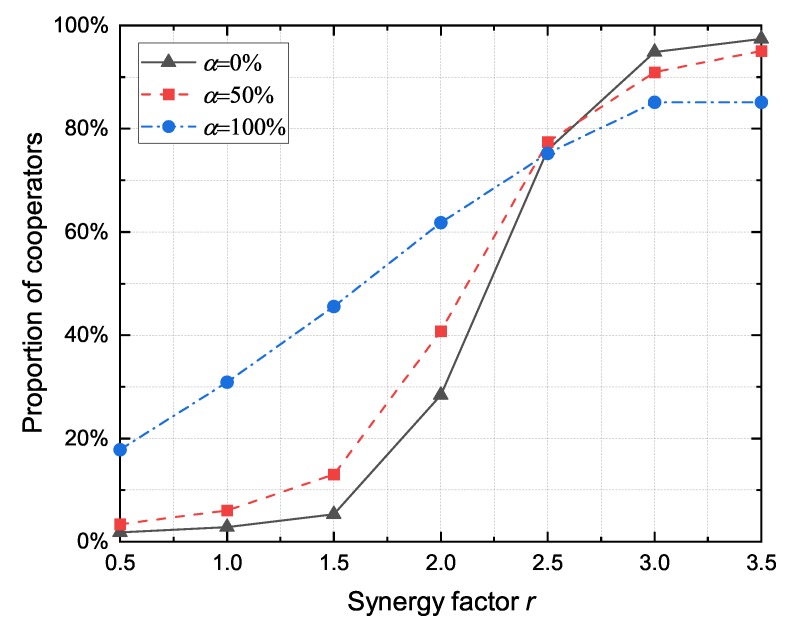
The proportion of cooperators under the different proportion of altruistic node α with respect to the synergy factor *r*.

**Figure 7:**
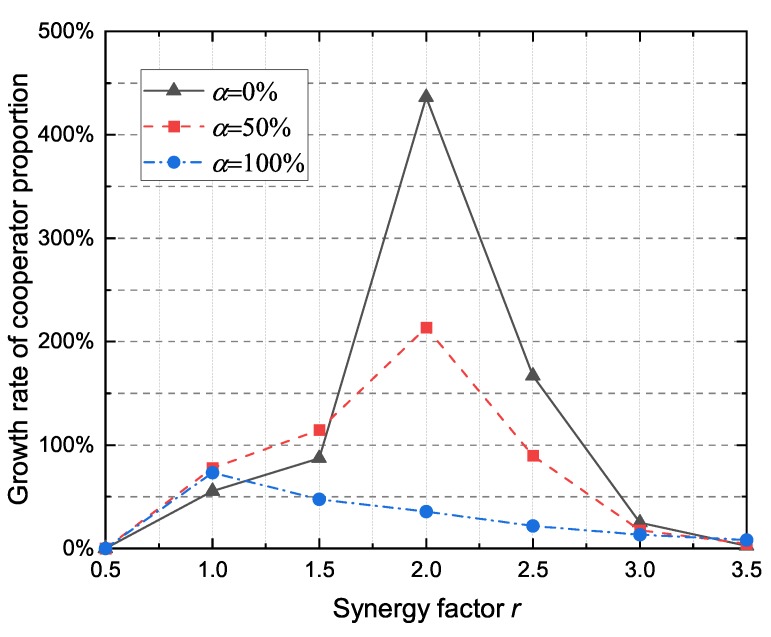
The growth rate of cooperator proportion under different altruistic node proportion α with respect to the synergy factor *r*.

As a heterogeneous network model, it is assumed that DAPGG contains only rational and altruistic nodes. However, when the network topology changes frequently, the proportion of each type of nodes is not constant. The results presented in Figure 6 show the impact of altruistic node proportion in the performance of the proposed model for different values of synergy factor *r*. When the synergy factor *r* is less than a certain threshold (*r* < 2.5), the proportion of cooperators increases with the increase of the altruistic nodes proportion. However, when the synergy factor exceeds the threshold (*r* > 2.5) the situation is reversed. As shown in Figure 7, the growth rate of the cooperator proportion fluctuates with the decrease of the proportion of altruistic nodes. In general, the introduction of altruistic nodes effectively increases the cooperator proportion and promotes the cooperator proportion to increase steadily with the change of synergy factor *r*. VANET has a continuously changing network topology, and nodes at the adjacent time instant usually have similar neighborhood structures. The altruistic node not only considers its own payoffs but also the benefits from the neighbors, which alleviates the fluctuation of the cooperator proportion caused by the growth of the synergy factor *r*.

#### 4.2.4. The Impact of Fitness Factor

**Figure 8:**
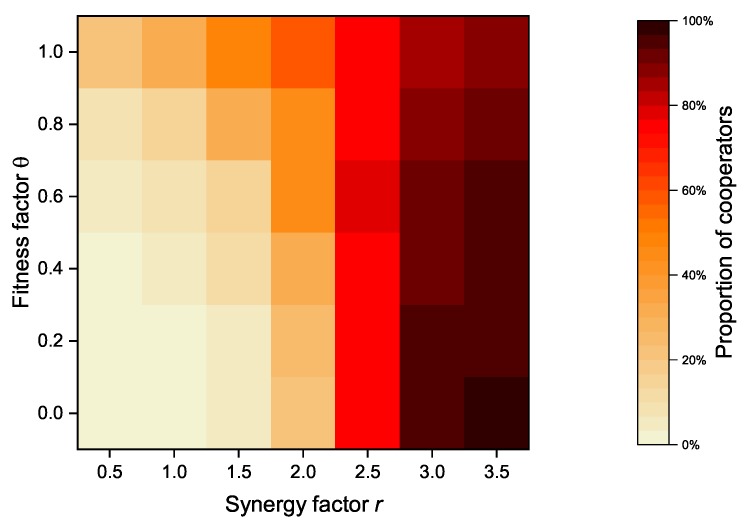
Relationship between fitness factor θ and synergy factor *r* of the model when the proportion of altruistic nodes is set to 50%.

**Figure 9:**
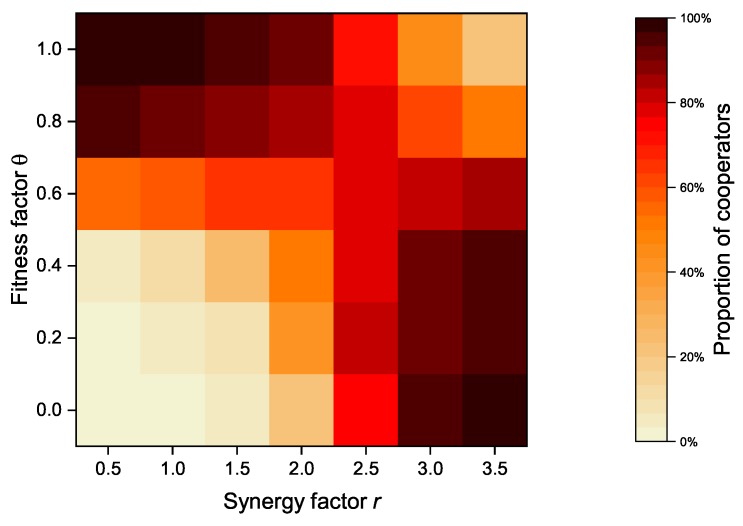
Relationship between fitness factor θ and synergy factor *r* of the model when the proportion of altruistic nodes is set to 100%.

In our proposed model, an altruistic node’s revenue is composed of its payoff and its neighbors’ payoffs. In case of strategy update, a node considers not only its own benefits but also its neighbor’s benefits. The payoff of its neighbors is proportional to the fitness factor θ. As an important parameter, Figure 8 shows the relationship between the fitness factor θ and the synergy factor *r* of the model when the altruistic nodes proportion is set to 50%, while Figure 9 shows the relationship between the fitness factor θ and the synergy factor *r* of the model when the altruistic nodes proportion is set to 100%. When the synergy factor *r* is at a low level (*r* < 2.5), the overall proportion of cooperators increases as the fitness factor *r* increases. However, when the synergy factor *r* exceeds a certain value (*r* > 2.5), the cooperator proportion decreases with the increase of the fitness factor, and vice versa.

As an important parameter of PGG, the synergy factor *r* is used to account for the synergistic effect of cooperation. The synergistic effect of cooperation refers to the fact that the benefits received as a result of cooperation may be greater than the sum of the contributed costs [32,33]. The synergy factor *r* is a measure of benefits obtained from the public pool, and an increasing synergy factor implies greater benefits received by individual nodes. In contrast to the synergy factor *r*, the fitness factor θ is the measure of the payoffs from its neighbors. Both of them can effectively increase the cooperator proportion under the certain conditions, but exceeding a certain threshold will actually bring the opposite effect. After all, the benefits received by the node and the payoff from neighbors are usually not balanced.

### 4.3. Zealots in DAPGG

#### 4.3.1. The Impact of Zealots Number

**Figure 10:**
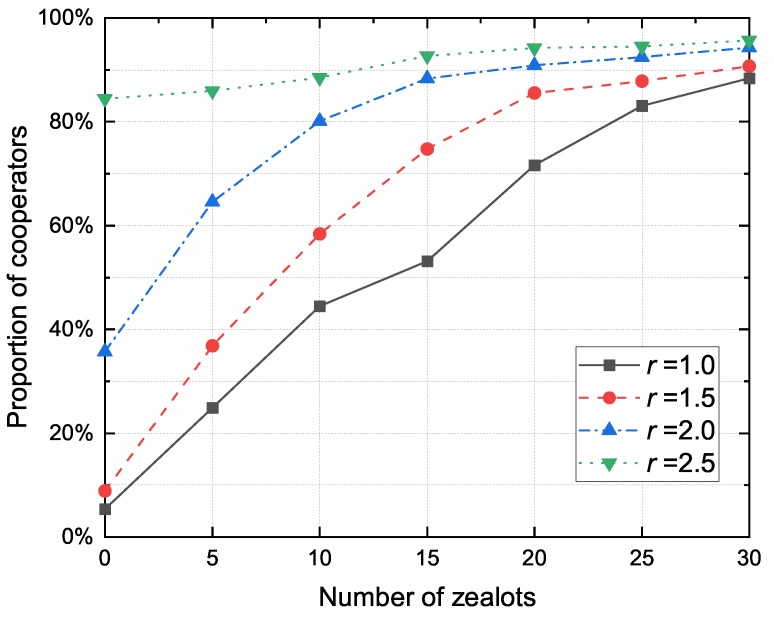
The proportion of cooperators to the synergy factor *r* for different zealot numbers.

As an important part of VANET, the RSU facilitates the communication between the vehicle and the transport infrastructure by transmitting data through various protocols. The RSU usually has abundant resources, so there is no need to worry about the resource burden caused by forwarding packets. In evolutionary games, this type of node is called Zealot. Figure 10 shows the proportion of cooperators to the synergy factor *r* for different zealot numbers. It can be observed that the proportion of cooperators increases with the number of zealots. On the one hand, when facing packets from their neighbors, zealots always maintain a cooperative strategy. Therefore, the more the zealots are, the higher the proportion of cooperators becomes. On the other hand, RSU only contributes to the public pool, but does not participate in the evolution of strategy. As the investment to the public pool is improved, RSU’s cooperative strategy may bring higher contributions to the public pool and speed up the process of packet forwarding. Thus, nodes are more likely to encounter cooperators holding new packets, leading to more chances of cooperative behavior.

#### 4.3.2. The Impact of Vehicle Number

**Figure 11:**
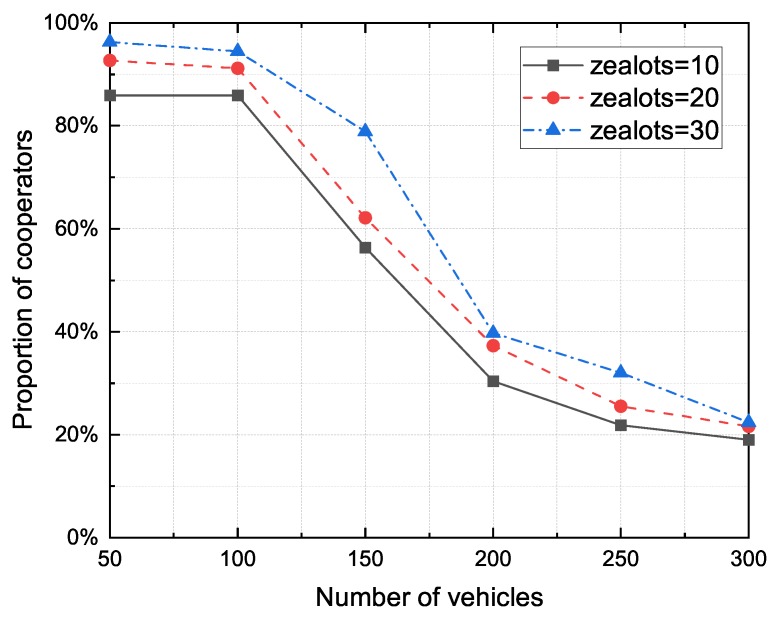
The proportion of cooperators to the zealot numbers for different number of vehicles.

With the introduction of the RSU, VANET can be regarded as a heterogeneous network composed of three types of nodes: rational nodes, altruistic nodes and zealots. We compare the cooperator proportion for different zealot numbers with respect to the number of vehicles in Figure 11. It can be seen that with the increase of network nodes, the overall cooperator proportion of the model would constantly decline. However, by increasing the number of zealots, it is possible to alleviate the decline in the cooperator proportion caused by the increase of vehicle numbers.

#### 4.3.3. The Impact of Altruistic Node Proportion

**Figure 12:**
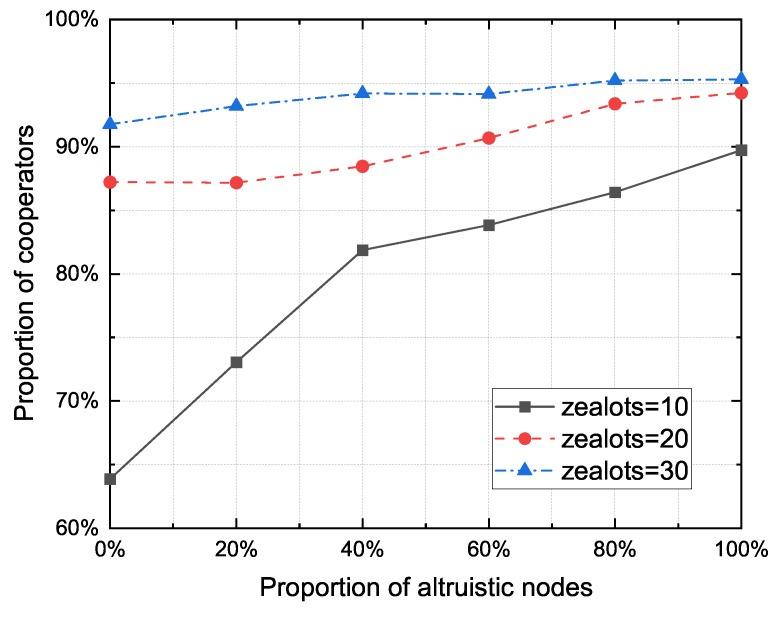
The proportion of cooperators to the zealot numbers for different altruistic node proportions.

Figure 12 shows the proportion of cooperators to the zealot numbers for different proportion of altruistic nodes. It can be observed that in the case of the same altruistic node proportion, the performance of cooperator proportion would be better when the number of zealots is high. Although the node density increases with the number of RSUs, the benefits of RSUs to the network are significantly greater than the negative effects of node density.

#### 4.3.4. The Impact of Fitness Factor

**Figure 13:**
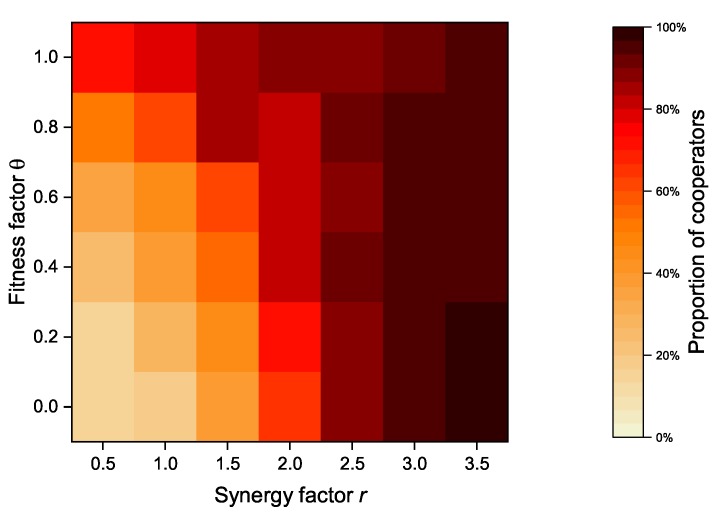
Cooperator proportion of the model for different synergy factors and different fitness factors when the zealots number is 10 and the proportion of altruistic nodes is set to 50%.

**Figure 14:**
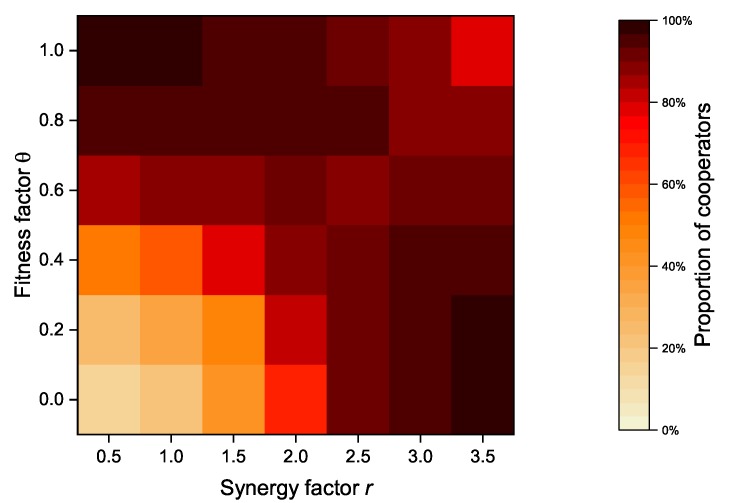
Cooperator proportion of the model for different synergy factors and different fitness factors when the zealots number is 10 and the proportion of altruistic nodes is set to 100%.

Figure 13 and Figure 14 show the relationship between the synergy factor *r* and the fitness factor θ after the introduction of zealots. Compared with the network without zealots (as shown in Figure 8 and Figure 9), the proportion of cooperators is significantly improved under the same synergy factor *r* and fitness factor θ. When the synergy factor *r* is small (*r* < 2.5), the proportion of cooperators increases with the increase of fitness factor θ, but when the synergy factor *r* is large (*r* > 2.5), the proportion of cooperators decreases slightly. Zealots greatly alleviate the downward trend of the proportion of cooperators in DAPGG as the fitness factor θ and the synergy factor *r* increase, even when the proportion of altruistic nodes is 100%.

## 5. Conclusions

In this paper, we propose a DAPGG model to explore the impacts of altruistic nodes and zealots on the cooperator proportion of VANET in heterogeneous networks. First, we separate the strategy and the attribute of nodes and then model VANET as a heterogeneous network of nodes with rational and altruistic attributes. Nodes with rational attributes only focus on their payoffs and maximize their payoffs by interacting with their neighbors, while nodes with altruistic attributes comprehensively consider the benefits of nodes in the neighborhood. Then, we introduce the Other-regarding Preferences (ORP) into the PGG-based VANET model to characterize the altruism and consider the neighborhood in the assessment of nodes more realistically. The results show that the introduction of altruistic nodes can effectively promote the proportion of cooperators to increase steadily with the synergy factor *r*. In addition, considering that VANET usually consists of vehicles and road-side units, we define road-side units as zealots that always maintain a cooperative strategy when facing the requests from neighborhoods. The results show that zealots alleviate the possible contradiction between the individual benefits measured by the synergy factor *r* and the neighborhood benefits measured by the fitness factor θ. Even when the synergy factor and the fitness factor are both high, the proportion of cooperators can be maintained at a considerable high level. In general, the introduction of altruistic nodes and zealots can significantly improve the proportion of cooperators, and the network can maintain a high level of cooperator proportion even under adverse conditions, thus providing a fresh overview and original perspective on the study of collaborative networks and their applications.

## Figures and Tables

**Table 1:** Comparison of DAPGG with several previous works.

Reference	Network	Game Model	Composition of Revenue	Application
[17]	Homogeneous	CG	Individual benefits	Message forwarding
[18]	Homogeneous	CG	Individual benefits	Advertisement distribution
[3]	Homogeneous	PGG	Individual benefits	Message dissemination
[4]	Homogeneous	PGG	Individual benefits	Group vehicular interaction
[9]	Homogeneous	PGG	Individual benefits	Content downloading
This paper	Heterogeneous	PGG	Individual, Neighborhood	Packet forwarding

**Table 2:** Simulation parameters.

Parameters	Values
Number of vehicles	[50, 100, 200]
Simulation map size	1000 m × 1000 m
Simulation time	800 s
Max velocity	20 m/s
Acceleration	1.0 m/s
Deceleration	3.0 m/s
Acceleration exponent	4
Desired time headway	1.5
Minimum gap	2
$R_{in}$	50 m
$R_{out}$	100 m
